# The value of RNA‐based platelet‐fluorescence testing in heat‐induced pseudothrombocytosis

**DOI:** 10.1002/jha2.417

**Published:** 2022-03-16

**Authors:** Malvika Gaur, Tushar Sehgal, Sudip Kumar Datta, Arulselvi Subramanian

**Affiliations:** ^1^ Department of Laboratory Medicine All India Institute of Medical Sciences New Delhi India

1

A 35‐year‐old gentleman presented to the hospital with a low‐grade fever for 5 days. The outpatient section of the annexe building adjacent to the main hospital received his EDTA‐anticoagulated venous blood specimen. On a Sysmex® XN‐series automated hematology analyzer, a complete blood count (CBC) revealed hemoglobin of 162 g/L, a leucocyte count of 8.98 × 10^9^/L, and an elevated platelet count of 779 × 10^9^/L. The RBC histogram had an abnormal height at the lower discriminator that did not start at the baseline (Figure 1A). Further research found that the automated platelet count using the fluorescence technique (PLT‐F) (Figure 1B) was 48% lower than the impedance‐based count (PLT‐I). RBC fragments were estimated to be 4.5% using optical measurements of platelets (PLT‐O) (Figure 1C) and RET channel (Figure 1D). Anisopoikilocytosis was evident on the blood film, with many tiny RBC fragments and microspherocytes (Figure 1E). The discrepancy in the platelet counts was attributed to the interference by RBC fragments. According to subsequent examination, the patient's blood sample arrived an hour later than the rest of the samples due to an unintended misplacement during transit. After excluding a mix‐up, a resampling was performed on the same day. The CBC revealed a normal platelet count of 401 × 10^9^/L and no RBC fragmentation. As a result, it was hypothesized that the prior blood specimen had been accidentally exposed to the hot (42°C) summer weather conditions in New Delhi. A number of factors influence platelet counts accuracy using automated hematology analyzers. Excessive RBC microcytosis or circulating red cell fragments, as observed in microangiopathic hemolytic anemia or severe burns, create pseudothrombocytosis, with a large amount of RBC slipping below the upper platelet threshold. A misleading increase in platelet count can be generated by in vitro red cell fragmentation caused by inadvertent heating of a blood sample. Similarly, due to size similarities with platelets, other nonplatelet particles, such as leukocyte cytoplasmic fragments in leukemia, bacteria, or fungi, may spuriously raise platelet count. Inadvertently, the PLT‐I and PLT‐O counts of such samples are recorded as platelets. The PLT‐F channel on the XN‐Series automated hematology analyzers uses a flow cytometry‐based platelet counting approach. Platelet RNA is labeled using fluorescent dyes, such as polymethine or oxazine, allowing nonplatelet particles to be excluded, while large or giant platelets can be counted. Gating separates the two populations (platelet and nonplatelet particles). Because microcytic red cells lack RNA, they do not absorb the dye, whereas reticulocytes, due to their larger size, are not a source of interference. As a result, the use of fluorescent‐based platelet testing on hematology analyzers may help to avoid misleading thrombocytosis diagnoses. The International Council for Standardization in Hematology has approved immunoplatelet analysis using platelet antigens CD41 and CD61 as the reference method; however, due to the need for a specialized analyzer and expensive reagents, it is challenging to use frequently in many Indian facilities.

**FIGURE 1:**
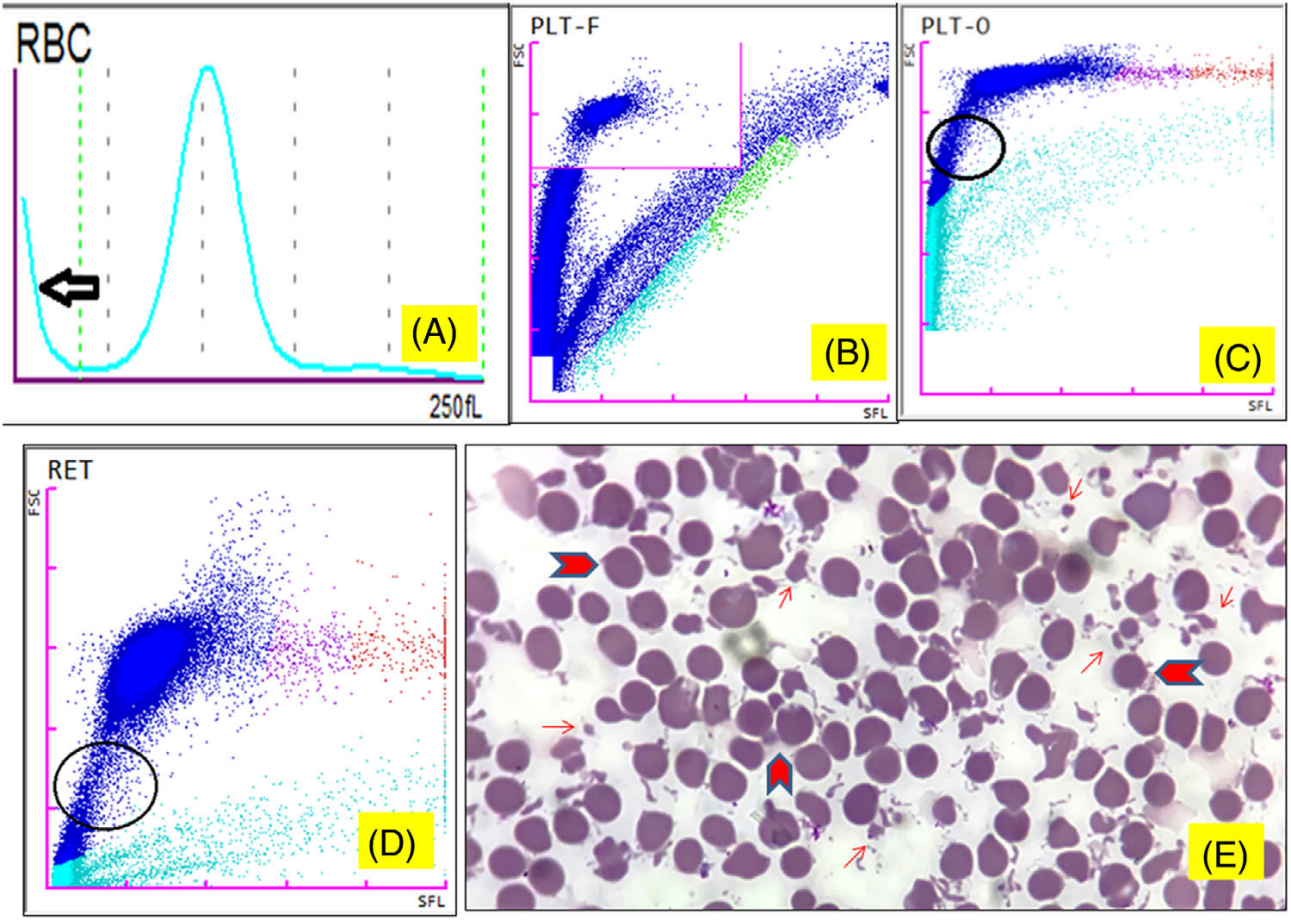
(A) An abnormal RBC histogram that does not begin at the baseline (left pointing arrow). (B) Automated platelet count using the fluorescence technique (PLT‐F). (C) Optical measurement of platelets (PLT‐O) showing RBC fragments (circle). (D) RET channel showing RBC fragments (circle). (E) Blood film shows anisopoikilocytosis with numerous tiny RBC fragments (arrow) and microspherocytes (small red blood cells with no central pallor) (shown by chevron)

## FUNDING

None to declare.

## CONFLICT OF INTEREST

The authors declare no conflict of interest.

## ETHICS STATEMENT

The patient's consent was obtained for the publication of the image.

